# Survival Benefits of Metformin for Colorectal Cancer Patients with Diabetes: A Systematic Review and Meta-Analysis

**DOI:** 10.1371/journal.pone.0091818

**Published:** 2014-03-19

**Authors:** Zu-Bing Mei, Zhi-Jiang Zhang, Chen-Ying Liu, Yun Liu, Ang Cui, Zhong-Lin Liang, Guang-Hui Wang, Long Cui

**Affiliations:** 1 Department of Colorectal Surgery, Xinhua Hospital, Shanghai Jiao Tong University School of Medicine, Shanghai, People's Republic of China; 2 Department of Epidemiology, School of Public Health, Wuhan University, Wuhan, People's Republic of China; University of Bari & Consorzio Mario Negri Sud, Italy

## Abstract

**Background:**

Several studies suggest that metformin has the potential effect of reducing cancer risk. However, its survival benefit in patients with colorectal cancer (CRC) and diabetes is unknown. The aim of our study is to address the effect of metformin on outcomes for CRC based on a systematic review and meta-analysis.

**Methods and Findings:**

We searched EMBASE and MEDLINE databases from inception through August, 2013, using search terms related to metformin, diabetes, colorectal cancer, and prognostic outcome. The outcome measures were hazard ratios (HRs) with 95% CIs comparing CRC survival in diabetic patients using metformin and without using metformin. The primary end points were overall survival (OS) and CRC specific survival (CS). A total of six cohort studies including 2,461 patients met full eligibility criteria. The pooled HR favoring metformin users was 0.56 for OS (95% CI, 0.41 to 0.77) and 0.66 for CRC-specific survival (95% CI, 0.50 to 0.87). Thus metformin therapy reduced the risk of all cause of death by 44% and the risk of CRC specific death by 34% in CRC patients compared to those in non-users. However, evidence of heterogeneity and possible publication bias was noted for OS.

**Conclusions:**

Patients with CRC and diabetes treated with metformin appear to have an improved survival outcome. Prospective study should be warranted to examine the association between metformin exposure intensity as well as some other confounding variables and survival outcome in diabetic CRC patients.

## Introduction

Previous epidemiologic studies suggest that patients with type 2 diabetes have a conspicuous increase in cancer risk and mortality compared with non-diabetic counterpart [1]–[6]. To our knowledge, several risk factors are shared by these two diseases, such as greater body mass index, smoking, poor eating habits and lack of exercise [3].

Although diabetes and its associated pathologic changes such as hyperinsulinemia, increase in C-peptide levels and chronic inflammation are likely major contributors to neoplastic process, several anti-diabetic drugs, including biguanide metformin, sulfonylureas, insulin and thiazolidinediones, have also been observed to affect cancer morbidity and prognosis [7]–[9]. Moreover, diabetic patients with cancer have worse prognosis and an increased risk of death [10]. Recent years have witnessed the focus on the research of metformin and CRC risk and outcomes with diabetes [11], [12].

Metformin, a first-line oral antidiabetic agent for type 2 diabetes, has been found to play a potential role in anticancer effect through molecular mechanisms of the ATM/LKB1/AMPK axis and mammalian target of rapamycin (mTOR)-signaling pathway [13]. The mTOR pathway regulates cell growth and tumorigenesis, and associates with tumor progression and prognosis. However, this role in CRC is mostly limited by observational population-based or clinic-based retrospective studies. No definitive conclusions on the effects of metformin for CRC can be drawn at the molecular level.

Up till now, the prognostic significance for diabetic patients with CRC using metformin in survival outcomes has not been systematically assessed. And recent meta-analysis only investigated the general cancer outcomes and all-cause mortality [10]. However, as a systemic disease which can involve different organs to various extent, diabetes can show variation between different cancer types on prognostic effects. Therefore, we performed a systemic review and meta-analysis to evaluate the effect of metformin on survival outcomes in diabetic patients with CRC.

## Methods

### Search Strategy

We searched the Ovid MEDLINE (1946 to August 2013) and EMBASE (1974 to August 2013) databases for relevant studies. MeSH terms combind with related text words and keywords were used. Our search terms included metformin (eg, “metformin”, “biguanides”), diabetes (eg, “diabetes mellitus,” “glucose intolerance,” “hyperglycemia”), colorectal cancer (eg, “colon/rectal/colorectal cancer/tumor/carcinoma/adenocarcinoma”) and prognostic outcome.(eg, “prognosis”, “survival”, “recurrence”, “mortality”, “outcome” “disease-specific survival”). No language restriction was performed. Hand searching was also performed on the reference lists of all included studies as well as reviews for additional relevant studies. We did not contact authors for unpublished relevant data.

### Eligibility Criteria

Eligible articles were considered in our study if they met the following criteria: original articles or abstract reported time to event data (hazard ratios [HRs] with 95% confidence interval [CI]) pertaining to association between CRC survival and metformin use. Diabetes was identified prior to colorectal cancer diagnosis based on medical or pathology reports. We excluded small sample size studies with low study quality or no time to event data provided. Furthermore, patients who were diagnosed CRC prior to diabetes or CRC cases identified at the time of death were excluded. Moreover, only patients with type 2 diabetes were included in our study.

### Data Extraction and Quality Assessment

Characteristics of each study were extracted by two reviewers independently (ZBM. and YL or ZLL) by scanning the title, abstract or full text. Disagreement was resolved by discussion or consensus or with a third reviewer (ZJZ). The following information was collected from eligible articles: authors, year of publication, study duration, exclusion criteria, number of patients with metformin use, patient age, stage of CRC, prognostic outcomes reported, use of multivariate models, adjustment factors and follow up period. STROBE checklist was applied for quality assessment in cohort studies concerning some of important factors recommended previously by Elm et al and Barone et al [10], [14].

### Statistical Analysis

The primary end point was overall survival (OS) defined as the time between the date of initial diagnosis of CRC and the date of death irrespective of cause of death. Colorectal cancer specific survival (CS), as the second end point, was defined as the interval between initial diagnosis of CRC and the last objective follow-up information or death caused by CRC.

Overall combined HRs and associated 95% CI were calculated using the DerSimonian-Laird method for a random-effects model as substantial interstudy heterogeneity existed for most outcomes [15]. To further investigate the robustness of the overall results, we performed sensitivity analyses by excluding one study each time and rerunning the analysis. Heterogeneity across studies was assessed by using the *I* ^2^ statistic with its values ranging from 0 to 100% and a value greater than 50% indicating substantial heterogeneity [16]. Potential publication bias was assessed by visually examining funnel plot asymmetry as well as Egger's test and Begg's test. If publication bias was detected, Duval and Tweedie nonparametric trim and fill method, used to estimate missing studies, was conducted for adjustment of the funnel plot and pooled HRs were recalculated [17]. All statistical analyses were conducted using Stata software (version 12.0; Stata Corporation, College Station, Tex).

## Results

### Literature Search

**Figure 1:**
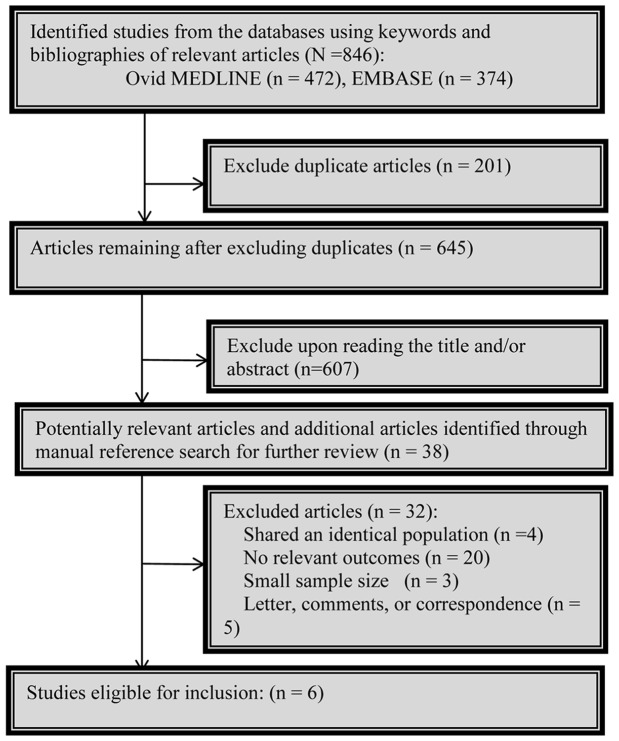
Flowchart of study selection.

Figure 1 shows the search process, which yielded a total of 846 citations using the search strategy (detailed in File S1), with 472 from OVID MEDLINE and 374 from EMBASE. After excluding 201 duplicate and 607 irrelevant articles based on abstracts or titles, we finally included 38 citations for detailed evaluation. Four original manuscripts provided estimates of HRs of CRC prognosis between metformin use and non metformin users [18]–[21]. And two additional studies were identified that had been published as abstracts from ASCO Annual Meeting [22], [23]. They were also included in the pooled analyses because sufficient prognostic data on key study variables could be abstracted. Therefore, a total of six eligible studies were left for our final analysis.

### Study Characteristics

Characteristics of the six selected studies are presented in Table 1. All were retrospective cohort studies that reported the hazard ratio (HR), a time to event outcome as the main interest, with six studies reporting OS and three CS. Several statistical methods were employed, including Kaplan-Meier survival analysis, multivariate Cox proportional hazard model or logistic regression model.

**Table 1:** Baseline characteristics of included studies in the meta-analysis.

			Total No. of CRC patients No of M users/ No of Non-users No./Total						
Authors and published years	Cohort Designation	Total No. in the Cohort	User %	None-users %	CRC Stage	Age (years)	Outcome measures	Survival analysis	Follow-up duration
GE Lee et al; 2012	Singapore National Cancer Centre study	1,455	219/344 64%	125/344 36%	II,III	NA	OS	Multivariate	Median 6.5 years
Bansal et al; 2011	VA cancer registry data base	5,052	141/571 25%	430/571 75%	NA	Median 64 (57–71)	OS	Univariate	Total 339 years of post-M follow-up. Total 6930 years of M-free follow-up
Garrett et al; 2011	MD Anderson Cancer Center study	4,758	208/424 49%	216/424 51%	All	Mean 62.7	OS	Multivariate	NA
Lee et al; 2012	Korean hospital based study .	6,108	258/595 43%	337/595 57%	All	Median 63 (30–88)	OS,CS	Multivariate	Median 41 months (range,1–119)
Cossor et al; 2013	WHI study	2,066	84/212. 40%	128/212 60%	All	Median DM+M 70 (52–84); DM-M 72 (56–88)	OS,CS	Multivariate	Median 4.1 years (range,3 day-14.4 years).
Spillane et al; 2013	National Cancer Registry Ireland study	3,816	207/315 66%	108/315 34%	I,II, III	Median DM+M 74 (71–80); DM-M 76 (71, 79)	OS,CS	Multivariate	≥4 years

Abbreviations: CRC  =  colorectal cancer; OS = overall survival; CS = cancer–specific survival; NA =  not recorded or available; DM = diabetes mellitus; M =  metformin; WHI =  Women's Health Initiative.

The selected studies were all published in recent three years (2011 to 2013), and the sample sizes of the cohorts ranged from 1,455 to 6,108, with a median of CRC cases with diabetes being 384 (range, 212 to 595). The percentage of metformin users in CRC patients ranged from 25% to 66%. Three studies were performed in the United States [18], [20], [23] and the other three were in Singapore [22], Korea [19] and Ireland [21]. One study involved only women [18] and the other five studies included both genders.

The STROBE checklist for cohort studies was used for quality assessment as was shown in Table 2 [24]. Five studies applied clinic-based cohort and the other one used population-based cohort. Four studies ascertained the diagnosis of diabetes or metformin exposure through medical records while others through interview, standardized questionnaires or registry data. Two studies used 9th or 10th edition of the International Classification of Diseases to ascertain the diagnosis of CRC or diabetes. All six included studies investigated metformin exposure as the primary prognostic variables. Adjusted models were applied in all the six studies, in which four were adjusted for age and CRC stage.

**Table 2:** Study quality assessment.

	Population Source		Diabetes and Metformin Exposure Ascertainment		Outcome Ascertainment		Diabetes Metformin exposure Evaluated As		
Reference	Population -Based Cohort	Clinic-Based Cohort	Medical Record/ Medication use	Other	Registry	Medical Record	Primary Exposure	One of Multiple Prognostic Factors	Statistical Analysis Adjusted Model?
GE Lee et al; 2012		Y	Y			Y	Y		Y
Bansal et al; 2011		Y		Y	Y		Y		Y
Garrett et al; 2011		Y	Y		Y	Y	Y		Y
Lee et al; 2012		Y	Y			Y	Y		Y
Cossor et al; 2013	Y			Y (interview or standardized questionnaires)	Y		Y		Y
Spillane et al; 2013		Y	Y	Y (tumour registration officers)	Y	Y	Y		Y

Abbreviations: Y, present in study.

### Meta-Analysis

**Figure 2:**
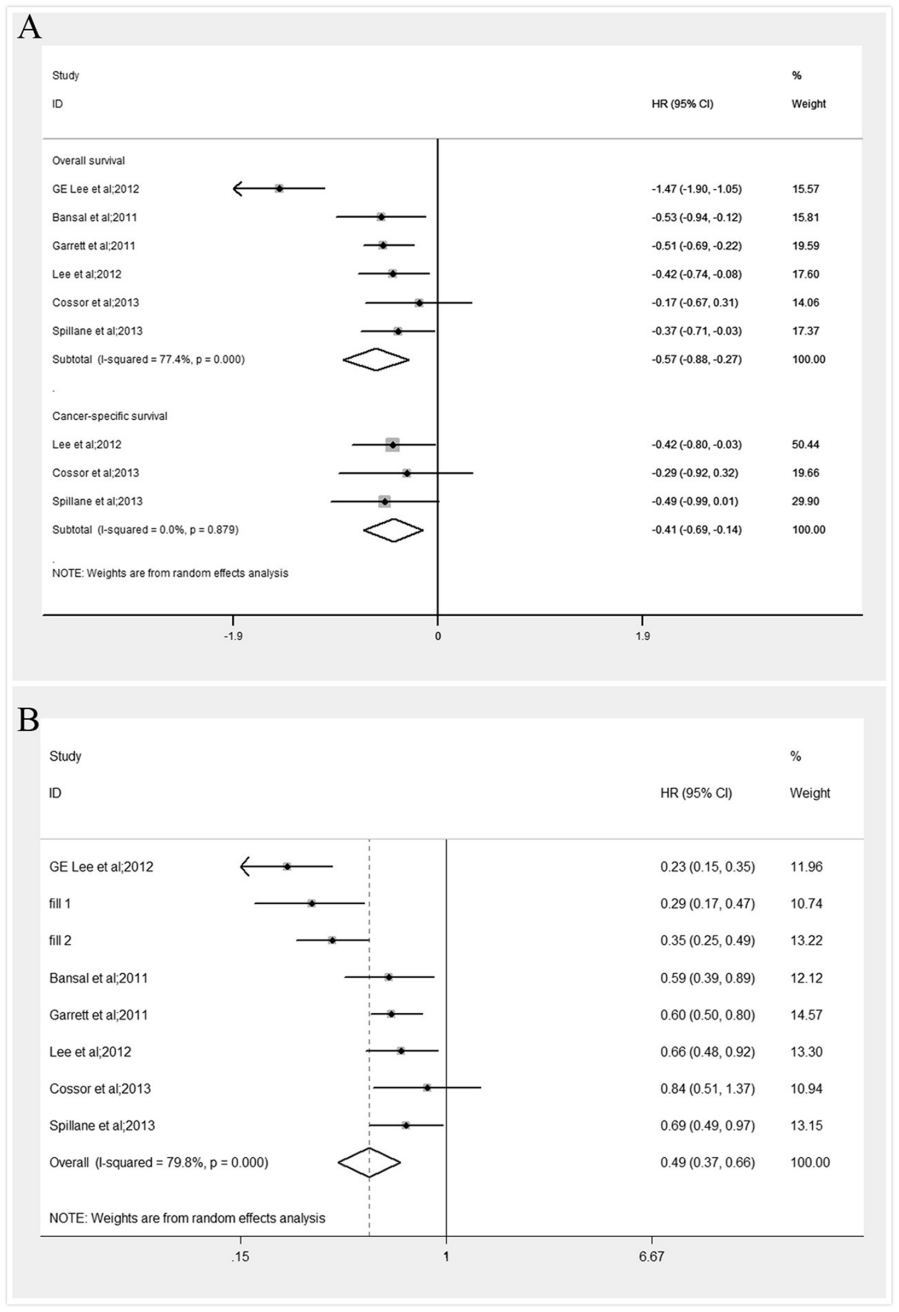
A. Meta-analysis of the effect of metformin use on survival outcomes for patients with colorectal cancer. **B.** Adjusted forest plot with hazard ratios (95% CI) for overall survival by trim and fill method.

**Figure 3:**
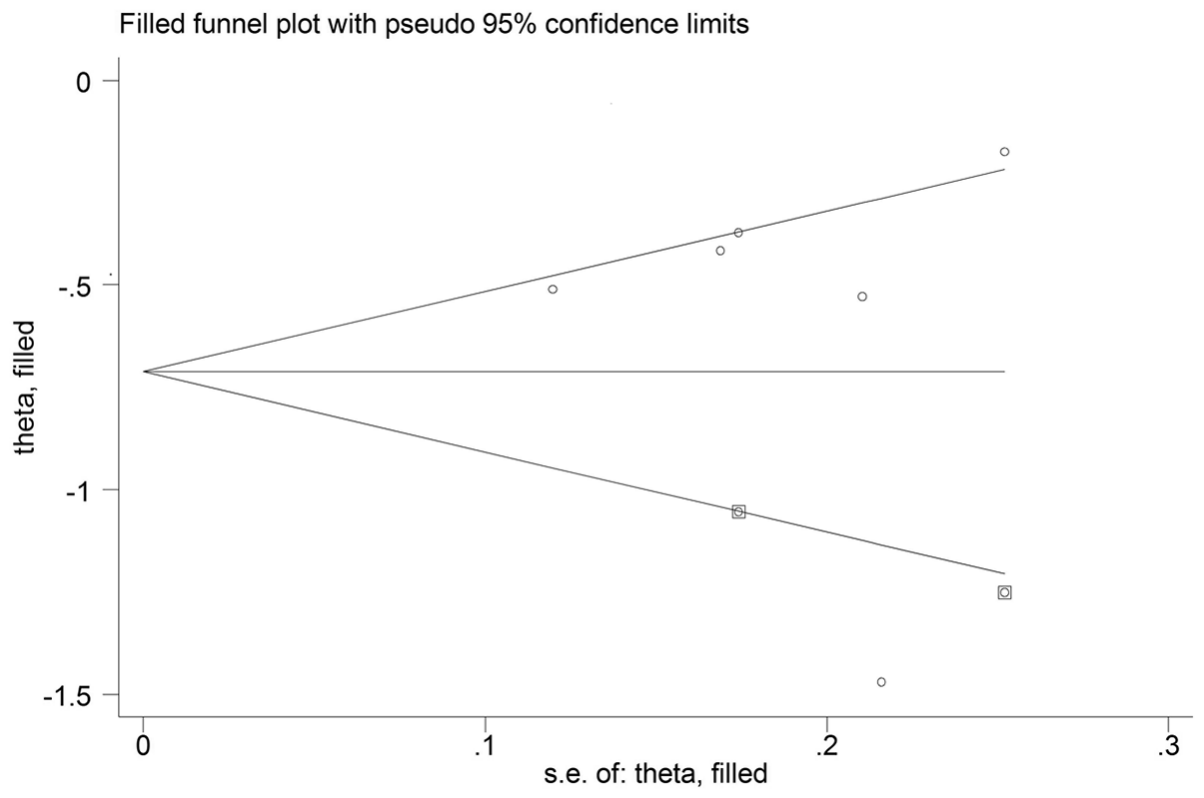
Plot of sensitivity analysis by excluding one study each time and the pooling estimate for the rest of the studies.

The estimated HRs for association between CRC survival and exposure to metformin for each study are shown in Figure 2A. The pooled results revealed an improved OS for metformin users compared with that for non-users (HR, 0.56; 95% CI, 0.41 to 0.77). Heterogeneity was present between the studies (I^2^ = 77.4% and P<0.001). Then we performed sensitivity analysis by excluding one study each time and recalculated the pooled HR for the remainder of the studies. It was found that none of the exclusions of a specific study would dramatically change the trend of our primary pooled results. However, the exclusion of one study by GE Lee et al [22] led to the relatively maximal change in pooled estimate visually (see Figure 3) and the recalculated HR was 0.65 (95% CI, 0.56 to 0.75), which did not alter the results materially.

Among the six selected studies, three also reported CRC-specific survival [18], [19], [21]. Lee et al found an increased colorectal cancer survival benefit by 34% in patients exposed to metformin compared with those who did not use metformin (median follow-up, 41 months; HR, 0.66; 95% CI, 0.45 to 0.98). Similar CRC-specific survival advantage was observed by Cossor et al (HR 0.78; 95%CI, 0.38 to 1.55) and Spillane et al (HR 0.44; 95% CI, 0.20 to 0.95) in diabetic CRC patients using metformin over non user.

### Publication Bias

**Figure 4:**
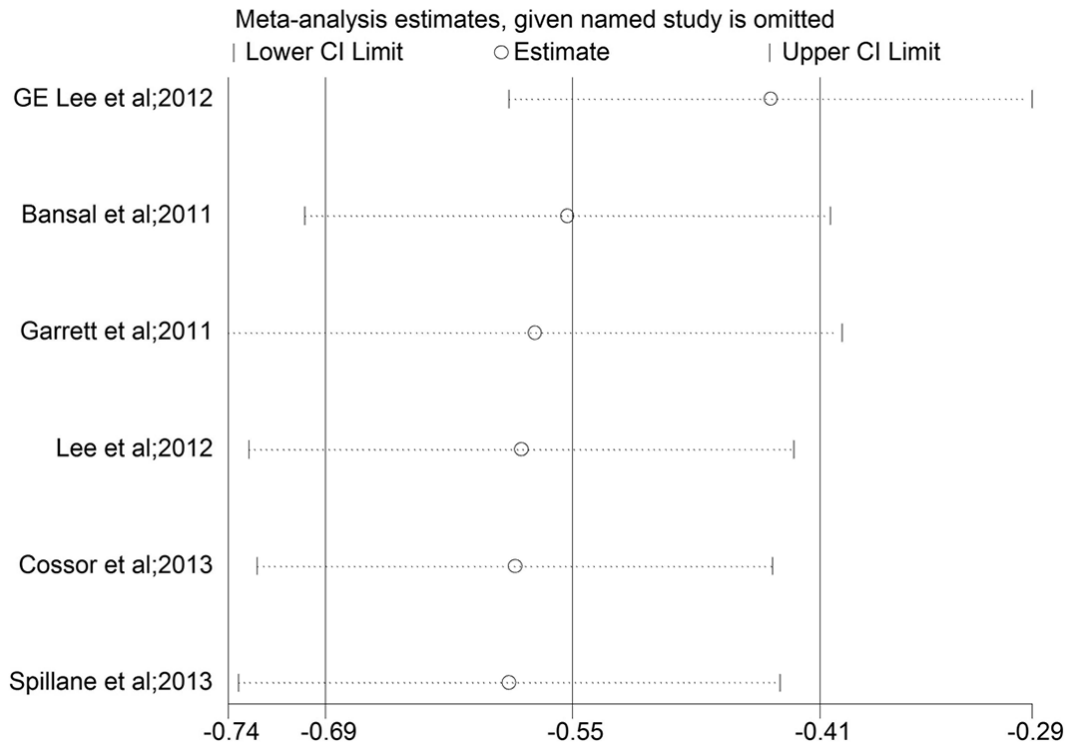
Funnel plots of the relationship between the HRs of individual studies and the precision of the study estimate (Log hazard ratio, horizontal axis;standard error, vertical axis).

**Figure 5:**
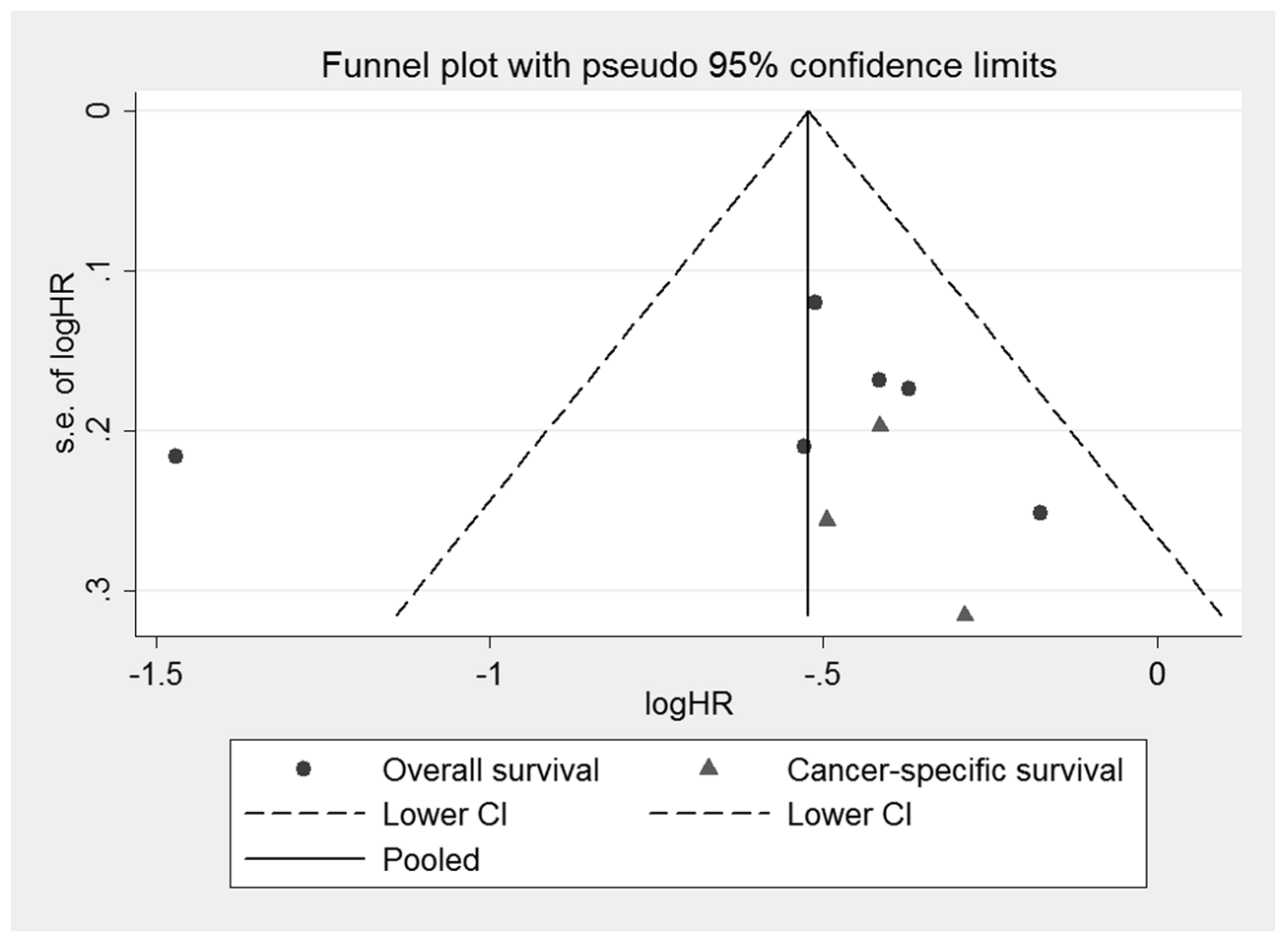
Filled funnel plot with 95% CI indicating two hypothesized studies missing.

We did not detect publication bias statistically based on the Egger's test (P = 0.73) or Begg's Test (P = 1.00). However, potential risk of bias was observed in the funnel plot (see Figure 4). Therefore, the trim and fill method was applied to further estimate the effect of publication bias, hypothesizing that two studies with unpublished results were missing using the random effect model (see Figure 5). And this approach resulted in an adjusted pooled HR of 0.49 (95% CI, 0.37 to 0.66). However, heterogeneity was still evident in this simulated meta-analysis (I^2^ = 79.8% and P<0.001) (see Figure 2B).

### Metformin Exposure and CRC Stage

Of three studies investigating the survival difference between metformin exposure and CRC cancer stage, Lee et al found that compared to non-metformin users, stage III CRC patients revealed a significantly higher CRC-specific survival rate (HR, 1.86; 95% CI 1.01 to 3.44; p = 0.048) and overall survival rate (HR, 1.72; 95% CI 1.01 to 2.94; p = 0.046) in metformin users. Garrett et al reported that with Cox proportional hazard models, poorer overall survival was indicated in stage III (HR, 1.60; 95% CI 1.02 to 2.40) and stage IV (HR, 5.60; 95% CI 3.70 to 8.40) CRC patients taking metformin than that in stage I and II. Moreover, Spillan et al observed that the inclusion of stage IV CRC patients could reduce the association between metformin exposure and CRC-specific survival.

### Metformin Exposure Intensity and CRC Survival

One study by Spillan et al investigated the association between metformin exposure and CRC survival. In this cohort with national prospectively-collected data, the risk of CRC-specific mortality was significantly lower in patients taking metformin exclusively at high dosing intensity (HR 0.44, 95% CI 0.20 to 0.95) compared to those not taking metformin. However, little difference was noted in associations between low and high dosing intensity metformin exposure and CRC-specific mortality in patients receiving non-metformin anti-diabetic drugs.

## Discussion

As the two most common diseases worldwide, diabetes and colorectal cancer (CRC) share many risk factors. Previous meta-analyses have demonstrated that type 2 diabetes is associated with increased risk of CRC [25], [26] and metformin is a commonly prescribed anti-diabetic agent in outpatients. We sought to comprehensively investigate the relationship between metformin exposure and CRC outcomes in patients with diabetes by pooling survival data from all studies. In fact, our meta-analysis including 23,255 participants from six cohort studies revealed that diabetic patients with CRC taking metformin achieved an estimated OS benefit of 44% compared with non-metformin users.

The potential antitumor effect for metformin has not been fully elucidated, although several observational studies have reported such a trend [12], [27]–[29]. Metformin mediates mammalian target of rapamycin (mTOR) pathway via the activation of AMPK and tuberous sclerosis complex 2 (TSC2), phosphorylation of TSC2 which leads to an inhibition of mTOR signaling and reduction in protein synthesis for cancer cells. It also promotes p53-dependent autophagy and cell cycle arrest through a decrease in cyclin D1 protein level [30]. Although experimental data indicate that metformin leads to mTOR inhibition, at present no mTOR inhibitors have been approved for the treatment of CRC patients.

Our meta-analysis might be a consistent finding among different population cohorts even though potential confounding factors and risk of publication bias do exist. Though the observational studies cannot be interpretated as causation, several aspects can be taken into consideration recommended by Hill et al as evidence to support our conclusion [31]. First, temporal association between metformin exposure and CRC incidence, which means CRC occurs after metformin exposure in all studies. Second, the results reveal a biological gradient or dose-response relationship in one study [21], with higher intensity metformin users exclusively having lower risk of CRC-specific mortality. Third, what we find is also biologically plausible as causality with the fact that metformin is found to reduce cancer cell proliferation, inhibit mTOR and protein synthesis and lead to cell cycle arrest. Fourth, consistent results are shown in the forest plot for both individual study and pooled estimates of the six included studies. Fifth, significant strength of the association between metformin users and non users is revealed for an estimated OS benefit of over 40%.

Several potential limitations to our meta-analysis need to be addressed. First, we did not fully investigate the heterogeneity of individual studies. Despite the random effects model being responsible for part of inter-study heterogeneity, variation of some other confounding factors in study design (eg, population source, methods of diabetes and metformin exposure ascertainment, outcome ascertainment, etc.) has also been ascribed to heterogeneity. Though adjusted models were used in most studies in our review, stratified analyses were not performed based on the adjusted variables due to limited number of studies. Second, some asymmetry in the funnel plot indicated publication bias. However, the pooled results obtained by the trim and fill method used to adjust the funnel plot asymmetry only slightly altered, which might suggest the robustness of our meta-analysis findings. Nevertheless, we still cannot fully exclude the potential possibility of some unpublished results based on this method. Third, the studies did not report the impact of date of diabetes onset or duration of metformin exposure on the CRC survival. Therefore, the observed benefit from duration of metformin use cannot be clearly defined. Fourth, other anti-diabetic drugs such as insulin and sulfonylureas were only adjusted in one study [19], which might inversely affect CRC survival. Finally, though two conference abstracts included containing detailed survival information for retrieval, some baseline characteristics could not be obtained for further stratified analysis.

One strength of our meta-analysis was the comprehensive search strategy for time to event survival data to evaluate the survival advantage of metformin users over non users in CRC patients. Thus, we were able to reasonably estimate the value of metformin for future clinical use. We also performed sensitivity analyses to investigate whether any particular study changed the results, but the findings were generally robust.

## Conclusions

In summary, our finding suggests that patients with CRC and diabetes treated with metformin appear to have an improved survival outcome. Further studies should be focused on prospective design to improve the study quality, as well as taking some confounding variables into consideration, such as date of diabetes onset, metformin exposure intensity and duration as well as other clinical characteristics. A strict follow up scheme is also warranted in future study design.

## Supporting Information

File S1 **Search Strategy S1–S2.** S1. Ovid MEDLINE(R) 1946 to Present with Daily Update S2. Embase Database.
(DOCX)

Checklist S1 **PRISMA checklist.**
(DOC)
