# Correction: Survival Benefits of Metformin for Colorectal Cancer Patients with Diabetes: A Systematic Review and Meta-Analysis

**DOI:** 10.1371/journal.pone.0103652

**Published:** 2014-07-25

**Authors:** The PLOS ONE Staff

The legends for Figures 3, 4, and 5 are incorrectly switched. The legend that appears for Figure 3 belongs with Figure 4, the legend that appears for Figure 4 belongs with Figure 5, and the legend that appears for Figure 5 belongs with Figure 3. The figures themselves appear in the correct order. Please see the complete, corrected Figure 3 here.

**Figure 3:**
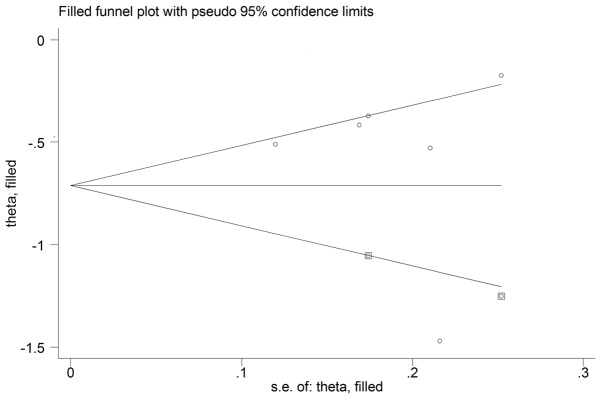
Filled funnel plot with 95% CI indicating two hypothesized studies missing.

Please see the complete, corrected Figure 4 here.

**Figure 4:**
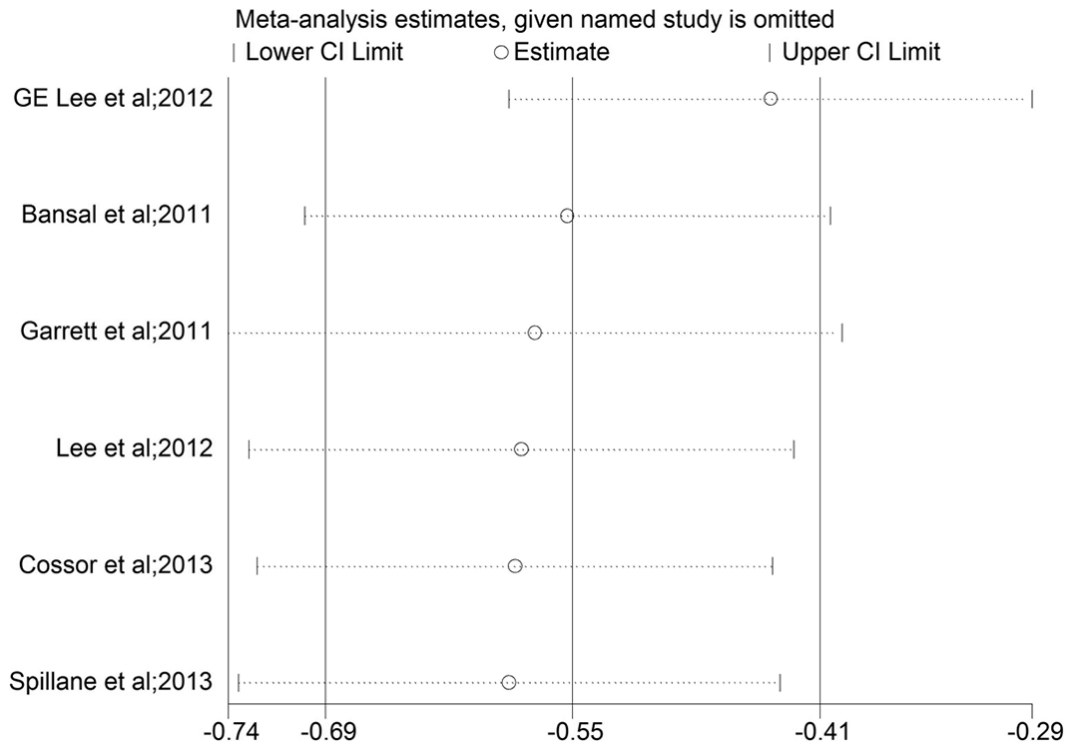
Plot of sensitivity analysis by excluding one study each time and the pooling estimate for the rest of the studies.

Please see the complete, corrected Figure 5 here.

**Figure 5:**
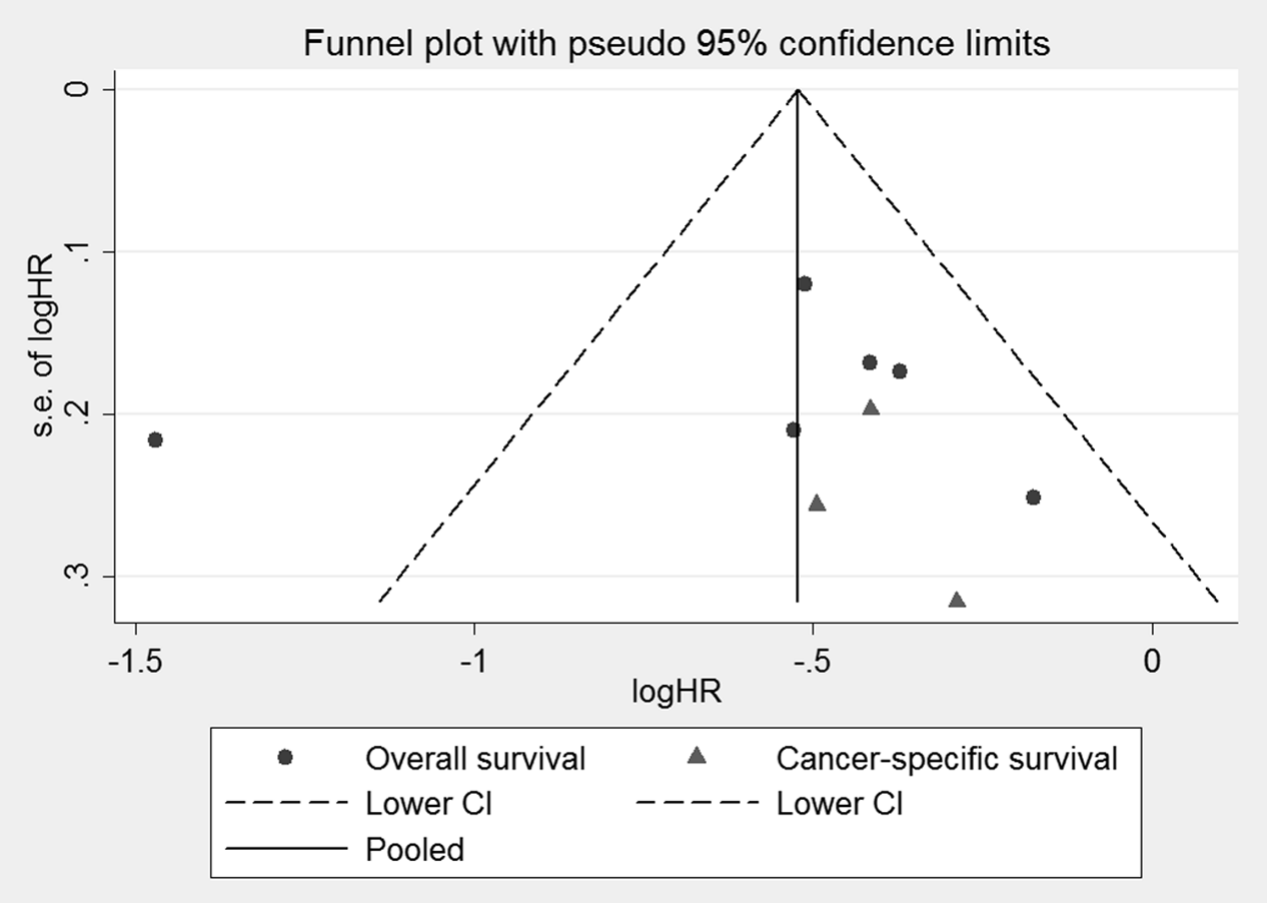
Funnel plots of the relationship between the HRs of individual studies and the precision of the study estimate (Log hazard ratio, horizontal axis;standard error, vertical axis).
