# Supplementary material for: Survival Benefits of Metformin for Colorectal Cancer Patients with Diabetes: A Systematic Review and Meta-Analysis
Source: PLoS One. 2014 Mar 19;9(3):e91818. doi: 10.1371/journal.pone.0091818 (PMC3960145; doi:10.1371/journal.pone.0091818)
Supplement: File S1 — Search Strategy S1–S2. S1. Ovid MEDLINE(R) 1946 to Present with Daily Update S2. Embase Database. (DOCX) [file pone.0091818.s001.docx]

**Search Strategy S1** Ovid MEDLINE(R) 1946 to Present with Daily Update

1. exp Colorectal Neoplasms/

2. (rectal or rectum or colon* or colorect* or colo-rect*).ti.

3. (cancer* or carcinom* or neoplas* or tumo* or adeno*).ti.

4. 2 and 3

5. 1 or 4

6. exp Mortality/

7. exp Survival Analysis/

8. exp Prognosis/

9. (prognos* or risk or survival or recurren* or mortality or predict* or outcome* or significan* or impact or detect* or relevan*).ti.

10. 6 or 7 or 8 or 9

11. exp Diabetes Mellitus/

12. (diabetes or diabetic or glycemic or glycaemic or "sugar*" or glucose intolerance or hyperglycemia).ab,ti.

13. 11 or 12

14. exp Metformin/

15. exp Biguanides/

16. (metformin* or glucophag* or biguanid*).ab,ti.

17. 14 or 15 or 16

18. 13 or 17

19. 5 and 10 and 18

**Search Strategy S2** Embase Database

1. 'colorectal tumor'/exp

2. rectal:ti OR rectum:ti OR colon*:ti OR colorect*:ti

3. cancer*:ti OR carcinom*:ti OR neoplas*:ti OR tumo*:ti OR adeno*:ti

4. #2 AND #3

5. #1 OR #4

6. prognos*:ti OR risk:ti OR survival:ti OR recurren*:ti OR mortality:ti OR predict*:ti OR

outcome*:ti OR significan*:ti OR impact:ti OR detect*:ti OR relevan*:ti

7. 'mortality'/exp

8. 'cancer mortality'/exp

9. 'cancer prognosis'/exp

10. #6 OR #7 OR #8 OR #9

11. 'diabetes mellitus'/exp

12. 'diabetes'/exp OR 'diabetic'/exp OR glycemic OR glycaemic OR 'sugar'/exp OR 'glucose intolerance'/exp OR hyperglycemia:ab,ti

13. 11 OR #12

14. 'metformin'/exp

15. 'biguanide derivative'/exp

16. metformin* OR glucophag* OR biguanid*:ab,ti

17. #14 OR #15 OR #16

18. #13 OR #17

19. #5 AND #10 AND #18
